# Novel Antioxidant, Deethylated Ethoxyquin, Protects against Carbon Tetrachloride Induced Hepatotoxicity in Rats by Inhibiting NLRP3 Inflammasome Activation and Apoptosis

**DOI:** 10.3390/antiox10010122

**Published:** 2021-01-15

**Authors:** Igor Y. Iskusnykh, Evgenii D. Kryl’skii, Darya A. Brazhnikova, Tatyana N. Popova, Khidmet S. Shikhaliev, Konstantin K. Shulgin, Larisa V. Matasova, Sergey S. Popov, Dmitry A. Zhaglin, Anastasia A. Zakharova, Nelli R. Popova, Nikolai Fattakhov

**Affiliations:** 1 Department of Anatomy and Neurobiology, University of Tennessee Health Science Center, Memphis, TN 38163, USA; 2 Department of Medical Biochemistry and Microbiology, Voronezh State University, Universitetskaya sq. 1, Voronezh 394018, Russia; evgenij.krylsky@yandex.ru (E.D.K.); dasha.brazhnikowa@yandex.ru (D.A.B.); biomed-popova@yandex.ru (T.N.P.); kkshulgin@mail.ru (K.K.S.); larissamatasova@yandex.ru (L.V.M.); dimitrizhaglin@yandex.ru (D.A.Z.); 3 Department of Organic Chemistry, Voronezh State University, Universitetskaya sq. 1, Voronezh 394018, Russia; shikh1961@yandex.ru; 4 Department of Organization of Pharmaceutical Business, Clinical Pharmacy and Pharmacognosy, Voronezh State Medical University Named after N.N. Burdenko, st. Studencheskaya 10, Voronezh 394036, Russia; popov-endo@mail.ru; 5 Department of Medical Biochemistry, Faculty of Biomedicine, Pirogov Russian National Research Medical University, Ostrovitianov St. 1, Moscow 117997, Russia; anzakh191@gmail.com; 6 Laboratory of Isotope Investigations, Institute of Theoretical and Experimental Biophysics, Russian Academy of Sciences, Pushchino 142290, Moscow Region, Russia; nellipopovaran@gmail.com; 7 Laboratory of Functional Materials Synthesis and Mineral Processing, Kurnakov Institute of General and Inorganic Chemistry, Russian Academy of Sciences, Moscow 119991, Russia; 8 Department of Biochemistry and Molecular Biology, University of Miami Miller School of Medicine, Miami, FL 33136, USA

**Keywords:** acute liver injury, carbon tetrachloride, ethoxyquin, deethylated ethoxyquin, oxidative stress, inflammasome, apoptosis, antioxidant enzymes, glutathione

## Abstract

Inflammation and an increase in antioxidant responses mediated by oxidative stress play an important role in the pathogenesis of acute liver injury (ALI). We utilized in silico prediction of biological activity spectra for substances (PASS) analysis to estimate the potential biological activity profile of deethylated ethoxyquin (DEQ) and hypothesized that DEQ exhibits antioxidant and anti-inflammatory effects in a rat model of carbon tetrachloride (CCl_4_)-induced ALI. Our results demonstrate that DEQ improved liver function which was indicated by the reduction of histopathological liver changes. Treatment with DEQ reduced CCl_4_-induced elevation of gene expression, and the activity of antioxidant enzymes (AEs), as well as the expression of transcription factors *Nfe2l2* and *Nfkb2*. Furthermore, DEQ treatment inhibited apoptosis, downregulated gene expression of pro-inflammatory cytokines (*Tnf* and *Il6*), cyclooxygenase 2 (*Ptgs2*), decreased glutathione (GSH) level and myeloperoxidase (MPO) activity in rats with ALI. Notably, DEQ treatment led to an inhibition of CCl_4_-induced NLRP3-inflammasome activation which was indicated by the reduced protein expression of IL-1β, caspase-1, and NLRP3 in the liver. Our data suggest that DEQ has a hepatoprotective effect mediated by redox-homeostasis regulation, NLRP3 inflammasome, and apoptosis inhibition, which makes that compound a promising candidate for future clinical studies.

## 1. Introduction

The liver is an important organ for systemic homeostasis and has a wide range of functions, including protein synthesis, production of bile, and detoxification of various metabolites. Toxicity caused by xenobiotics, including drugs, can cause derangement in any of the liver functions and potentially lead to acute liver injury (ALI) associated with high rates of morbidity and mortality [1,2]. Carbon tetrachloride (CCl_4_)-induced hepatotoxicity in rats is a common experimental model in which CCl_4_ treatment can produce oxidative stress leading to other forms of liver damage. This model mimics human xenobiotic-induced ALI, for which no effective therapy has been developed yet.

CCl_4_ is metabolized by liver isoenzyme cytochrome P450 2E1 to highly reactive trichloromethyl free radicals, which initiate lipid peroxidation and destruction of cell [3,4]. A major defense mechanism against CCl_4_-induced reactive oxygen species (ROS) generation involves the antioxidant enzymes (AEs) such as superoxide dismutase (SOD), catalase (CAT), glutathione peroxidase (GPX), and glutathione S-transferase (GST) that convert ROS into non-toxic more stable molecules. The activities of GPX and GST depend on the availability of reduced glutathione (GSH) that is maintained by enzyme glutathione reductase (GR). The expression of key components of the GSH antioxidant system as well as enzymes involved in nicotinamide adenine dinucleotide phosphate (NADPH) regeneration, ROS, and xenobiotic detoxification is regulated by the transcription factor nuclear factor erythroid-2-related factor 2 (Nrf2) (encoded by *Nfe2l2*) [5]. During oxidative stress and subsequent antioxidant response, Nrf2 rapidly translocates from the cytoplasm to the nucleus where its activation takes place. Recent studies have noted a key protective role of Nrf2 activation in attenuating ALI [6,7]. In addition, the deficiency of Nrf2 in hepatocytes sensitizes the cells to CCl_4_-induced oxidative damage and inflammatory response [8].

The inflammation response is also an important event for CCl_4_-induced ALI [9]. The NOD-like receptor protein 3 (NLRP3) inflammasome is a multiprotein complex consisting of NLRP3, apoptosis-associated speck-like protein containing a C-terminal caspase recruitment domain, and caspase-1 controlling the release of pro-inflammatory interleukin-1 beta (IL-1β). The NLRP3 inflammasome has been reported to be involved in the pathogenesis of ALI [7,10]. ROS-induced activation of the NLRP3 inflammasome leads to pyroptotic and apoptotic cell death, but only pyroptosis, a pro-inflammatory form of programmed cell death, is unique to the inflammasome [11,12]. Accumulating evidence indicates that hepatocyte pyroptosis has an important role in various inflammation-related liver diseases including alcoholic hepatitis and steatohepatitis [13]. The CCl_4_-induced formation of ROS by damaged hepatocytes may activate the nuclear factor κB (NF-κB) transcription factor family that plays critical role in the inducible expression of inflammatory genes during the inflammatory response. Synthesis of the major hepatotoxicity mediators, cytokines tumor necrosis factor-α (encoded by *Tnf*) and interleukin-6 (encoded by *Il6*), mainly by Kupffer cells is mediated by NF-κB, as is the expression of pro-inflammatory mediator cyclooxygenase 2 (encoded by the *Ptgs2*) [14,15]. The non-canonical or “alternative” signaling NF-κB pathway predominantly targets activation of the NF-κB2 (p52) protein and its precursor p100 (encoded by *Nfkb2*). Initially thought to be mostly related to lymphocyte development and adaptive immunity, the non-canonical NF-κB pathway has also recently been reported to contribute to the development of ALI and fibrosis [16,17,18] and, therefore, is to be considered as a plausible drug target.

Implementation of antioxidant substances exhibiting anti-inflammatory and anti-apoptotic activities may be an effective therapeutic strategy for the prevention and treatment of ALI [19,20,21]. This could be attributed to investigating the effects of the ethoxyquin (1,2-dihydro-6-ethoxy-2,2,4-trimethylquinoline) derivatives developed as antioxidants for the potential treatment of pathologies implicating ALI [22]. Ethoxyquin is a synthetic antioxidant that is included in some animal and human foods to prevent the oxidation of lipids and lipid-soluble components [23]. Studies have shown that dietary supplementation with ethoxyquin can provide cellular protection against the toxic effects of a variety of environmental chemicals in the livers of brown bullhead catfish by induction of GST activity, as well as protect rat liver cell membranes against NADPH/iron-induced peroxidation via modulation GSH levels [24,25]. Other studies have reported that ethoxyquin exhibits anti-inflammatory activity and an ability to partially prevent the hepatotoxic effects of CCl_4_ [26,27,28]. Our previous study indicated lipid peroxidation inhibition properties of deethylated ethoxyquin (DEQ) [29]. However, other beneficial effects of DEQ, as well as the possible mechanisms whereby DEQ mediates these beneficial effects on CCl_4_-induced ALI need to be further investigated. Thus, the aim of our present study was to explore the role of DEQ in CCl_4_-induced ALI focusing on its effect on inflammatory responses, antioxidant systems and apoptosis.

## 2. Materials and Methods

### 2.1. Chemicals

**Figure 1:**
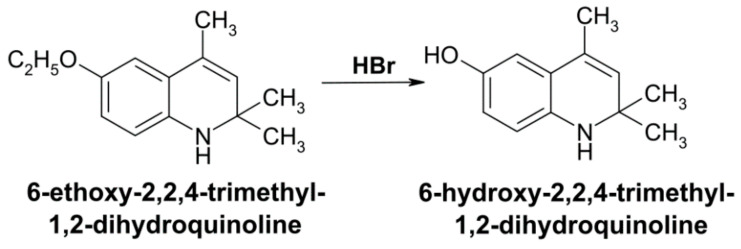
Synthesis of 6-hydroxy-2,2,4-trimethyl-1,2-dihydroquinoline by dealkylation of 6-ethoxy-2,2,4-trimethyl-1,2-dihydroquinoline when exposed to hydrobromic acid.

The synthesis of DEQ (6-hydroxy-2,2,4-trimethyl-1,2-dihydroquinoline) was performed by the dealkylation of ethoxyquin exposed to hydrobromic acid [30] (Figure 1). The structure of DEQ was confirmed by nuclear magnetic resonance spectroscopy and chromatography-mass spectrometry. Phenazine methosulfate (PMS), reduced and oxidized forms of glutathione, nicotinamide adenine dinucleotide (NADH), and NADPH were purchased from AppliChem GmbH (Germany). Trichloroacetic acid (TCA), 5,5′-Dithiobis(2-nitrobenzoic acid) (DTNB), 1-chloro-2,4-dinitrobenzene (CDNB), 3,3′,5,5′-tetramethylbenzidine (TMB), hydrogen peroxide, and GR were purchased from Sigma-Aldrich (USA). Nitroblue tetrazolium (NBT) chloride was purchased from Alfa Aesar (UK).

### 2.2. Biological Activity/Toxicity Prediction

The biological activity of DEQ was evaluated using the PASS (Prediction of Activity Spectra for Substances) software [31]. The PASS program allows for the estimation of the probable profile of biological activity of a drug-like organic compound based on its structural formula and for analysis of the structure-activity relationships for a broad training set entailing more than 300,000 compounds with known biological activities. As a result, the PASS program provides a list of biological activity types for which the probability to be revealed (P_a_) is calculated and varies from zero to one. If 0.5 < P_a_ < 0.7, the corresponding compound is likely to exhibit a particular activity in experiments. If P_a_ > 0.7, the studied compound is very likely to reveal activity in experiments.

The toxicity class and median lethal dose (LD_50_) for DEQ were predicted in silico using ProTox-II webserver that incorporates pharmacophore binding models, fragment propensities, and machine-learning models [32]. The ProTox-II server classifies the analyzed compound into one of six Globally Harmonized System of Classification and Labelling of Chemicals categories: class 1 includes compounds with LD_50_ values ≤5 mg/kg (fatal if swallowed), class 2 includes compounds with 5 < LD_50_ ≤ 50 mg/kg (fatal if swallowed), class 3 includes substances with 50 < LD_50_ ≤ 300 mg/kg (toxic if swallowed), class 4 includes substances with 300 < LD_50_ ≤ 2000 mg/kg (harmful if swallowed), class 5 includes substances with 2000 < LD_50_ ≤ 5000 mg/kg (may be harmful if swallowed), and class 6 includes safe chemicals (LD_50_ > 5000 mg/kg).

### 2.3. Animals and Experimental Design

Forty-eight male Wistar rats, 4–6 months old and weighing 200–250 g, were housed normal light-dark conditions (12:12) for the entire experiment and given ad libitum access to food and water ad libitum. The animals were randomly divided into the following groups: control, DEQ, CCl_4_, and CCl_4_ + DEQ (*n* = 12 in each group). The control group received 1 mL of Vaseline oil via oral gavage. In the DEQ group, rats were treated with 50 mg/kg DEQ once per day for 4 days. The animals in the CCl_4_ group received a single intragastric dose of CCl_4_ (0.064 mL dissolved in 1 mL of Vaseline oil/100 g body weight). The rats in CCl_4_ + DEQ group received DEQ (50 mg dissolved in 1 mL of 1% soluble starch solution/1 kg body weight) 3 h after CCl_4_ administration every 24 h for 4 days. All animals were humanely euthanized on day 4 to harvest blood and livers. Thus, the animals received a dose of DEQ comparable to the daily human dose of the hepatoprotector silymarin based on the rat body weight. The DEQ was administered after the induction of pathology daily until the animals were removed from the experiment. The study was approved by the Institutional Animal Care and Use Committee of The Voronezh State University (Voronezh, Russia).

### 2.4. Caspase Activity Assessment

The enzyme activities of caspase-8 and caspase-3 were measured using colorimetric assay kits purchased from BioVision (Milpitas, CA, USA). The assay kits detected caspase activation by spectrophotometric determination of *p*-nitroaniline produced through hydrolysis of such substrates as acetyl-Ile-Glu-Thr-Asp *p*-nitroaniline and acetyl-Asp-Glu-Val-Asp *p*-nitroanilide by caspase-8 and caspase-3, respectively. The fluorescence emission of *p*-nitroaniline was detected at a wavelength of 405 nm.

### 2.5. Aminotransferase Activity Assessment

Serum activities of aspartate aminotransferase (AST) and alanine aminotransferase (ALT) were analyzed using a Hitachi U1900 spectrophotometer (Minato-ku, Japan) with the commercial diagnostic kits (Olvex Diagnosticum, St. Petersburg, Russia) according to the manufacturer’s instructions.

### 2.6. Histopathological Examination

The liver tissues were fixed in 10% formalin in normal saline solution, embedded in paraffin and sectioned at a thickness of 8 μm on the Thermo Scientific HM 325 microtome. Sections were deparaffinized using xylene, mounted on standard glass slides and stained with hematoxylin and eosin (HE). HE-stained sections were observed for any histopathological abnormalities under a light microscope. At least three different sections were analyzed per liver sample.

### 2.7. Immunofluorescent Staining

The livers tissues were post-fixed in fresh 4% paraformaldehyde overnight at 4 °C and cryoprotected with 30% sucrose solution for 24 h. Histological sections (thickness 16 μm) of paraformaldehyde-fixed liver samples were treated with a serum-free protein block reagent (Agilent Technologies, Santa Clara, CA, USA) and incubated with primary antibodies against NLRP3 (AdipoGen Life Sciences, San Diego, CA, USA), caspase-1 (AdipoGen Life Sciences, San Diego, CA, USA) and IL-1β (Cell Signaling Technology, Danvers, Massachusetts, USA) overnight at 4 °C in a humidified chamber. Slides were washed in phosphate buffered saline (PBS), incubated with secondary anti-mouse antibodies for 1 h, mounted with ProLong Diamond reagent (Invitrogen, Carlsbad, CA, USA) for visualization by the Leica TCS SP5 laser scanning microscope (Leica Microsystems, Wetzlar, Germany). Negative controls were run by omitting the primary antibody step.

### 2.8. Myeloperoxidase Activity Assay

MPO enzyme activity was estimated indirectly by the oxidation of TMB as previously described by Suzuki et al. [33]. According to this method, 10 μL of sample was added to 110 μL of a TMB solution and 80 μL of 0.75 mM hydrogen peroxide. After 5 min of incubation at 37 °C, the reaction was terminated by adding sulfuric acid solution, and the amount of reaction product was determined spectrophotometrically at 350 nm.

### 2.9. Measurements of Antioxidant Enzyme Activity

The liver tissues were homogenized with a T10 homogenizer (IKA-Works, Staufen im Breisgau, Germany) in fourfold volume of homogenization buffer consisting of 1 mM ethylenediaminetetraacetic acid (EDTA), 1% β-mercaptoethanol, and 0.5 M Tris-HCl buffer (pH 7.8). The homogenates were centrifuged at 8000× *g* for 15 min, and the supernatants were used for the assays. All enzymatic assays were carried out using a Hitachi U1900 spectrophotometer (Minato-ku, Japan) in duplicate.

SOD activity was measured by the indirect method developed by Nishikimi et al. based on the reduction of NBT [34]. In this assay, SOD competes for the superoxide radicals generated in the presence of PMS and NADH reducing reduction of NBT. The reaction mixture consisted of 0.33 mM EDTA, 0.1 M phosphate buffer (pH 7.8), 0.8 mM NADH, 0.41 mM NBT, and 0.01 mM PMS. The absorbance of formed blue formazan was measured at 540 nm.

CAT activity was assessed according to the spectrophotometric assay developed by Goth [35]. The reaction mixture contained 0.08% hydrogen peroxide in 0.1 M Tris-HCl buffer (pH 7.4) as a substrate. The decomposition of hydrogen peroxide by CAT was terminated by adding 4.5% ammonium molybdate. The intensity of the formed yellow complex was measured at 410 nm.

GPx activity was assayed according to Paglia and Valentina [36]. Hydrogen peroxide (0.37 mM) and GSH (0.85 mM) were used as substrates and coupled oxidation of NADPH (0.12 mM) by GR (1 U/mL) was determined at a wavelength of 340 nm.

GR activity was evaluated by measuring the oxidation of NADPH (0.16 mM) using oxidized glutathione as a substrate [37]. The reaction mixture contained 50 mM potassium phosphate buffer (pH 7.4), 0.8 mM oxidized glutathione, 0.16 mM NADPH, and 1 mM EDTA.

GST activity was assessed by the method of Warholm et al. using GSH and CDNB as substrates [38].

### 2.10. Measurement of Reduced Glutathione Concentration

GSH concentration was assessed by the reaction with DTNB, resulting in a yellow colored product measurable at 412 nm [39]. The liver tissue homogenates in 0.1 M phosphate buffer (pH 7.4) and serum samples were acidified by the addition of 20% TCA and centrifuged at 3000× *g*. The GSH concentration was measured in the supernatant mixed with phosphate buffer and DTNB (96% ethanol for control samples) using a Hitachi U1900 spectrophotometer (Minato-ku, Japan).

### 2.11. Quantitative PCR

Total RNA was extracted using ExtractRNA kit (Eurogen, Moscow, Russia) according to the instructions of the manufacturer. Quantitative PCR (qPCR) was performed using an amplifier ANK-32 (Syntol, Moscow, Russia) with qPCRmix-HS SYBR kit (Eurogen, Moscow, Russia). Sequences of qPCR primers used to evaluate expression of genes related to the inflammation (*Nfkb2*, *Il1b*, *Il6*, *Tnf*, and *Ptgs2*), antioxidant defense system (S*od1*, *Cat*, *Gpx1*, *Gsr*, *Gsta2*, *Nfe2l2*, and *Foxo1*), and apoptosis (*Casp3*, *Casp8*, *Bax*, *Bcl2*, and *Aifm1*) are represented in the Appendix A. Relative expression levels of genes were normalized to a reference *Gapdh* gene. qPCR data were depicted in the heat map view using BAR HeatMapper Plus Tool. 

### 2.12. Statistical Analysis

Statistical analysis was performed using IBM SPSS Statistics for Windows, Version 25 (IBM Corporation, USA). All experimental data were represented as mean ± standard deviation (SD) and were examined for statistically significant differences using one-way analysis of variance (ANOVA) with Tukey’s multiple comparisons test. Differences between experimental groups were considered as statistically significant when the *p* value was < 0.05.

## 3. Results

### 3.1. Toxicological Properties and Biological Activity Profile of DEQ

At the initial stage of the study, we utilized in silico PASS analysis to estimate the potential biological activity profile of 6-hydroxy-2,2,4-trimethyl-1,2-dihydroquinoline as a candidate against CCl_4_-induced hepatotoxicity. Screening of hepatoprotective properties of DEQ using the PASS program revealed that DEQ has 20 different types of predicted biological activities with P_a_ values > 0.5. Table 1 shows the biological activity spectra predicted using the PASS computer program. The prediction of toxicity demonstrated that DEQ belongs to toxicity class 4 for acute oral toxicity with a predicted LD_50_ value of 1450 mg/kg. Thus, it is considered to be safe to use (Table 1). Additionally, two different toxicity targets including amine oxidase A (average pharmacophore fit 34.97%) and the progesterone receptor (average similarity to known ligands 76.7%) were predicted with probable binding.

The predicted reductant activity of DEQ corresponds to the highest value of P_a_. Therefore, the reducing power of DEQ may therefore serve as a significant indicator of its potential antioxidant activity. It is interesting to note, that DEQ, which is predicted to be a lipid peroxidase inhibitor, shows anti-inflammatory and free radical scavenger types of activity which seems to be beneficial for the ameliorating an acute liver injury and potentially explains the results of our study.

### 3.2. DEQ Alleviates CCl_4_-Induced Histological Changes in the Liver

**Figure 2:**
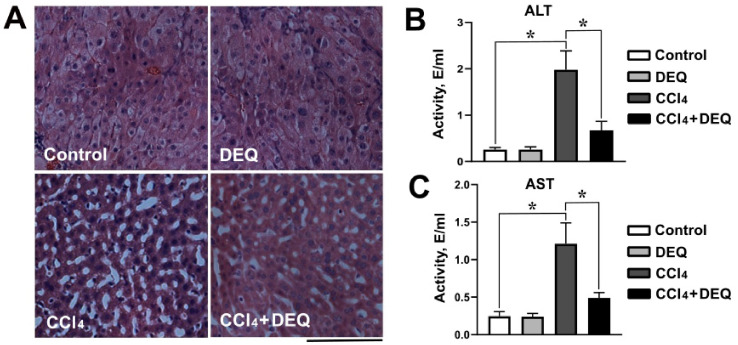
Effect of DEQ administration on hepatocellular damage in rats with CCl_4_-induced liver injury. (**A**). Representative images of hematoxylin and eosin (H&E) stained sections of the liver showing normal tissue in DEQ group, large necrosis regions in CCl_4_ group and restored tissue architecture in CCl_4_ + DEQ group. (**B**,**C**). ALT (B) and AST (C) serum activities were significantly increased in CCl_4_ group compared to the control group. Treatment with DEQ 3 h after CCl_4_ administration every 24 h for 4 days restored ALT and AST serum activities in rats. * *p* < 0.05. All data are expressed as mean ± SD. Scale bar: 100 μm.

To evaluate hepatoprotective effects of DEQ treatment during the CCl_4_-induced ALI, we used serum ALT and AST activities, and hepatic histopathological examination as indicators of liver injury. As we demonstrated in Figure 2A, hepatic histopathological examination of the DEQ treated control group revealed no degenerative signs. It corresponded to the normal liver tissue of the control group (no treatment) where most hepatocytes were normal with acidophilic cytoplasm and vesicular nuclei. In contrast to the control group, CCl_4_-treated group demonstrated severe hepatocellular damage characterized by large regions of a necrosis and significant loss of tissue architecture. DEQ treatment of rats with CCl_4_-induced ALI reduced the severity of liver tissue necrosis and alterations in liver tissue architecture. 

As represented in Figure 2B,C, CCl_4_ administration increased serum ALT and AST activities, in comparison with the control group (*p* < 0.05), whereas treatment with DEQ reduced CCl_4_-induced elevation of serum ALT and AST activities. 

### 3.3. DEQ Inhibits CCl_4_-Induced Inflammatory Responses via NLRP3 Inflammasome Pathway in the Liver

**Figure 3:**
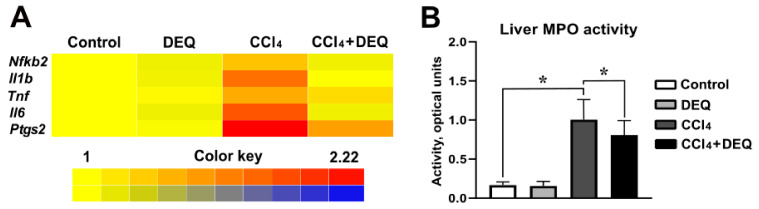
Effect of DEQ administration on hepatic *Nfkb2*, *Il1b*, *Tnf*, *Il6*, and *Ptgs2* mRNA levels, and myeloperoxidase (MPO) activity in rats with CCl_4_-induced liver injury. DEQ treatment downregulated expression of pro-inflammatory genes (*Nfkb2,* *Il1b*, *Il6*, *Tnf*, and *Ptgs2)* (Figure 3A, *p* < 0.05), and decreased myeloperoxidase (MPO) activity in rats with CCl_4_-induced liver injury (Figure 3B, * *p* < 0.05). Data are expressed as mean ± SD.

Previous studies have defined that CCl_4_-induced hepatotoxicity contributes to the development of the inflammatory response by triggering the gene expression of the major pro-inflammatory mediators such as NF-κB2, IL-6, IL-1β, TNF-α, and cyclooxygenase 2 [40]. To evaluate whether DEQ treatment inhibits liver inflammation caused by CCl_4_ administration, the expression of the aforementioned genes in the liver was measured. There was no difference between control and DEQ-treated control animal groups. However, in comparison with rats from the control group, rats with CCl_4_-induced ALI showed significantly (*p* < 0.05) elevated expression of *Nfkb2*, *Il1b*, *Il6*, *Tnf*, and *Ptgs2* mRNAs (Figure 3A). DEQ treatment significantly (*p* < 0.05) downregulated CCl_4_ -induced elevation of expression of these genes in the liver (Figure 3A).

Moreover, treatment with DEQ significantly (*p* < 0.05) reduced CCl_4_-dependent increase in MPO activity (Figure 3B), which indicates that DEQ treatment ameliorates CCl_4_-associated liver neutrophil infiltration.

**Figure 4:**
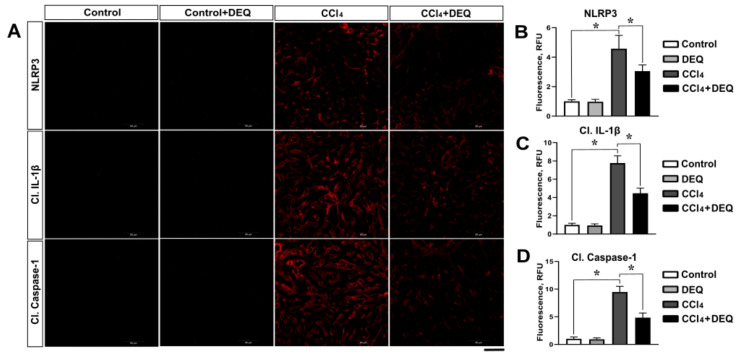
Effect of DEQ administration on NLRP3 inflammasome activation and subsequent pyroptosis with liver damage induced by CCl_4_. Representative microphotographs (**A**) and quantification of NLRP3 (**B**), cleaved IL-1β (**C**), and cleaved caspase-1 (**D**) across the groups. The NLRP3, IL-1β, and caspase-1 expression levels were significantly increased in in CCl_4_ group compared to the control group. Treatment of rats with DEQ 3 h after CCl_4_ administration every 24 h for 4 days significantly reduced NLRP3 inflammasome activation determined by the reduced protein expression of NLRP3, cleaved caspase-1, and IL-1β. The level of NLRP3, IL-1β, and caspase-1 proteins in DEQ group remained relatively unchanged compared with the control group. * *p* < 0.05. All values are expressed as fold change ± SD. Scale bar: 50 μm.

Activation of NLRP3 inflammasome contributes to liver injury through the induction of the pro-inflammatory cytokines and the effects of caspase-1 mediated pyroptosis [41] The main components of NLRP3 inflammasome assembly as well as the consequent production of IL-1β and caspase-1 were analyzed to measure the level of inflammasome activation in rat liver. Our data revealed that the expression of NLRP3, IL-1β, and caspase-1 were significantly (*p* < 0.05) increased in rats with ALI compared to control group (Figure 4A–D). Treatment of rats with DEQ markedly decreased the CCl_4_-induced activation of NLRP3 inflammasome determined by the reduced protein expression of NLRP3, cleaved caspase-1, and IL-1β (Figure 4A–D). The level of NLRP3, IL-1β, and caspase-1 proteins in DEQ-treated control group remained relatively unchanged compared with the control group.

### 3.4. DEQ Attenuates CCl_4_-Induced Hepatic Apoptosis

**Figure 5:**
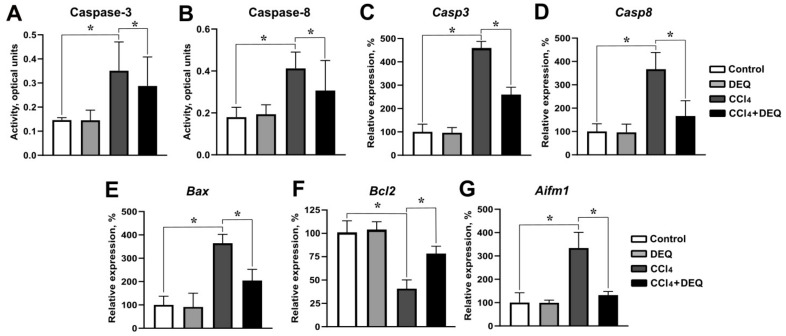
Effect of DEQ administration on hepatic apoptosis in rats with CCl_4_-induced liver injury. The caspase-3 (**A**) and caspase-8 (**B**) activities were significantly increased in the liver of animals from CCl_4_ group compared to the control group. CCl_4_ administration resulted in a significant increase in the *Casp3*, *Casp8*, *Bax*, and *Aifm1* mRNA levels (**C–E**,**G**), while the expression of *Bcl2* was significantly decreased (**F**). Treatment of rats with DEQ 3 h after CCl_4_ administration every 24 h for 4 days significantly reduced CCl_4_-induced caspase-3 (**A**) and caspase-8 (**B**) activities in the liver of rats as well as *Casp3, Casp8, Bax,* and *Aifm1* mRNA levels (**C**–**E**,**G**). Treatment with DEQ significantly increased CCl4-reduced expression of *Bcl2* (**F**). * *p* < 0.05. Activity values expressed as optical units per milligram of protein. All data are expressed as mean ± SD.

To determine the effect of DEQ on CCl_4_-induced apoptosis, we next measured the gene expression of *Casp3*, *Casp8*, *Bax, Bcl2*, and activities of caspase-3 and caspase-8 in hepatic tissue of rats. Our studies revealed that ALI induction was accompanied by a more than two-fold increase in the activities of caspase-3 and caspase-8 in rat liver (Figure 5A, B). DEQ treatment significantly (*p* < 0.05) decreased CCl_4_-induced elevation of caspase-3 and caspase-8 activities (Figure 5A,B). qPCR analysis revealed that CCl_4_ administration led to a significant increase in the expression of proapoptotic genes such as *Casp3*, *Casp8*, and *Bax* (Figure 5C–E, *p* < 0.05), while the mRNA level of antiapoptotic protein-*Bcl2* was significantly decreased compared to the control group (Figure 5F, *p* < 0.05). DEQ treatment of rats with ALI led to a reduction of CCl_4_-induced expression of *Casp3, Casp8,* and *Bax* (Figure 5C–E, *p* < 0.05). Treatment with DEQ significantly increased CCl_4_-reduced expression of *Bcl2*, which indicates its anti-apoptotic effect (Figure 5F, *p* < 0.05). No differences in expression of *Casp3*, *Casp8*, *Bax*, *Bcl2*, and caspase-3 and caspase-8 activities were observed between control and DEQ-treated control groups of animals.

To study whether apoptosis during CCl_4_-induced liver injury is related to mitochondrial apoptosis signaling pathway, we next measured the expression of mitochondrial intermembrane space protein, AIF, in liver tissue [42]. The results showed that an increase in *Aifm1* mRNA expression during CCl_4_-induced ALI was significantly (*p* < 0.05) reduced by DEQ (Figure 5C). Thus, DEQ promotes the inhibition of mitochondrial apoptosis pathway signaling, which might at least partly determine its hepatoprotective effect.

### 3.5. DEQ Restores the Activities of Antioxidant Enzymes and Reduced Glutathione Concentration in CCl_4_-Induced Liver Injury

**Figure 6:**
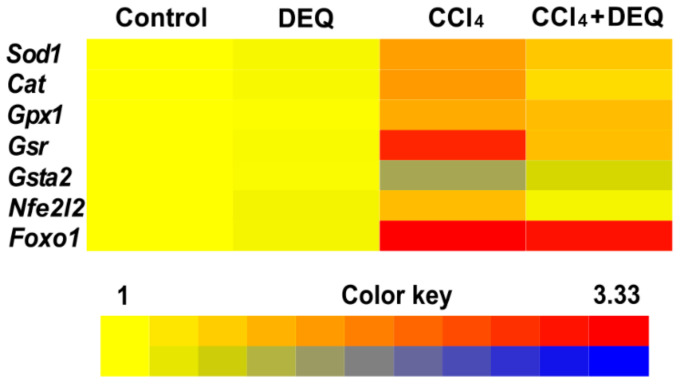
Analysis of hepatic S*od1*, *Cat*, *Gpx1*, *Gsr*, *Gsta2*, *Nfe2l2*, and *Foxo1* mRNA levels by qPCR across the groups (*p* < 0.05). Treatment with DEQ reduced CCl4-induced elevation of gene expression (S*od1*, *Cat*, *Gpx1*, *Gsr,* and *Nfe2l2*) in the liver of rats. Data are depicted in the heat map view in logarithmic scale.

Recent studies highlighted that ROS-dependent expression regulation of antioxidant system during CCl_4_-induced hepatotoxicity is mediated by Nrf2 and Foxo1 transcription factors [43,44]. To study whether DEQ treatment is able to provide expressional regulation of genes involved in the maintenance of redox homeostasis such as S*od1*, *Cat*, *Gpx1*, *Gsr*, *Gsta2*, *Nfe2l2*, and *Foxo1*, the expression of these genes was determined by qPCR analysis. As represented in Figure 6, rats with CCl_4_-induced ALI showed significantly (*p* < 0.05) elevated expression of S*od1*, *Cat*, *Gpx1*, *Gsr*, *Foxo1*, and *Nfe2l2* genes, whereas expression of *Gsta2* was significantly (*p* < 0.05) downregulated compare to the control group. DEQ treatment significantly (*p* < 0.05) downregulated CCl_4_-induced elevation of expression of S*od1*, *Cat*, *Gpx1*, *Gsr*, and *Nfe2l2* genes, but did not cause significant changes in the expression of *Foxo1*. Expression of *Gsta2* was significantly (*p* < 0.05) downregulated in CCl_4_-treated group of rats. However, DEQ treatment of rats with ALI led to upregulation of *Gsta2* expression with respect to CCl_4_-treated group (*p* < 0.05).

**Figure 7:**
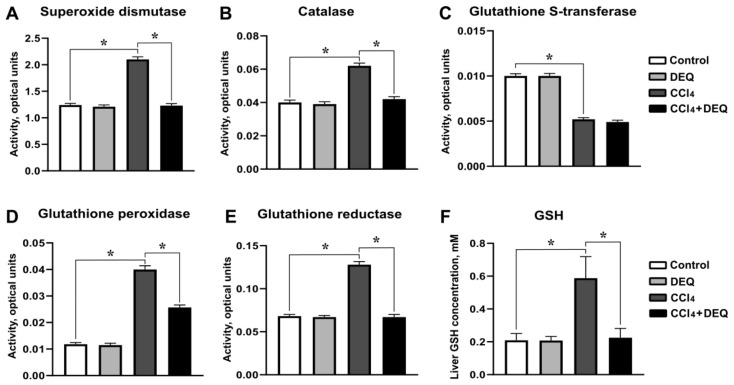
Effect of DEQ administration on liver activities of antioxidant enzymes and reduced glutathione in rats with CCl4-induced liver injury. Treatment of rats with DEQ 3 h after CCl_4_ administration every 24 h for 4 days significantly reduced CCl_4_-induced liver activities of superoxide dismutase (**A**), catalase (**B**), glutathione peroxidase (**D**), glutathione reductase (**E**), and reduced glutathione (**F**). The activity of glutathione transferase (**C**) was significantly increased in the liver of animals from CCl_4_ group compared to the control group. * *p* < 0.05. Activity values expressed as enzyme activity per milligram of protein. All data are expressed as mean ± SD.

We further examine the liver activities of AEs such as SOD, CAT, GPX, GSR, and GST in rats after CCl_4_ administration. There was no difference in activities of the aforementioned enzymes between control and DEQ-treated control animal groups. In comparison with rats from control group, CCl_4_ administration caused increase in liver activities of SOD, CAT, GPX, and GSR enzymes (*p* < 0.05), whereas activity of GST was significantly reduced (Figure 7A–E, *p* < 0.05). DEQ treatment significantly reduced CCl_4_-induced liver activities of SOD, CAT, GPX, and GSR (*p* < 0.05), while, activity of GST was not significantly affected compared to CCl_4_-treated group (Figure 7A–E). Notably, DEQ treatment significantly (*p* < 0.05) reduced CCl_4_-dependent elevation of GSH concentration in the liver (Figure 7F). These findings suggest, that DEQ improves the maintenance of liver redox homeostasis in CCl_4_-dependent ALI. 

## 4. Discussion

This study is the first, to our knowledge, to show the hepatoprotective properties of DEQ derivatives mediated by the inhibition of NLRP3-inflammasome assembly and restoration of redox homeostasis of the liver tissues. The principal findings of our study are as follows: (I) DEQ treatment of rats with CCl_4_-induced ALI reduces the severity of the pathological process in liver, i.e., tissue necrosis, alterations in liver architecture, and enhanced hepatocyte volume; (II) DEQ administration prevents CCl_4_-induced increase in the expression and activity of antioxidant enzymes in the liver and downregulates the expression of several transcription factors regulating oxidative stress and inflammation, such as NF-κB2, and Nrf2; and (III) DEQ induces a decrease in the intensity of CCl_4_-induced apoptosis and inflammation in the liver by inhibiting NLRP3-inflammasome functioning. Therefore, DEQ possesses hepatoprotective properties and inhibits two crucial pathogenetic pathways of ALI, such as intensification of oxidative stress and activation of NLRP3-inflammasome. Thus, the studied dihydroquinoline derivative ameliorates disease progression and can be considered a new approach for pathogenetic therapy of ALI.

Drug-induced ALI is a common disorder and potential adverse reaction to practically all classes of medication [45]. Peroral administration of CCl_4_ to rodents is a well-known model of toxic liver damage. CCl_4_-induced severe hepatotoxicity is mediated by several cellular and molecular mechanisms including the activation of lipid peroxidation and immune response in the liver cells. Preclinical and clinical studies emphasize the importance of oxidative stress as one of the main pathogenic pathways of ALI [46]. CCl_4_ metabolism mediated by CYP2E1 is followed by the generation of ROS, which might cause hepatocyte cytolysis. ROS generation during CCl_4_-induced intoxication, particularly CCl_3_^•^ and CCl_3_OO^•^, promotes lipid peroxidation and antioxidant imbalance in the liver resulting in tissue damage [47]. In our study, CCl_4_ administration was shown to be followed by an increase in the activities of antioxidant enzymes, such as SOD, catalase, GP, and GR as well as in a rise in GSH concentration in the liver of rats. These changes in the antioxidant system observed after CCl_4_ treatment might be considered an adaptive response to the developing liver injury. Moreover, the enhanced expression of genes of the antioxidant enzymes in rat liver induced by CCl_4_ might be associated with an increase in the Nrf2 expression, which is particularly sensitive to an altered redox state [48]. Previous studies show that ROS disable the negative regulator of this transcription factor Keap1, thus causing Nrf2 translocation to the nucleus. Nuclear Nrf2 binds to AREs and activates the transcription of ARE-driven genes encoding detoxification and antioxidant enzymes [49,50]. In addition to the enhanced *Nfe2l2* mRNA expression, our study showed an increased expression of the gene encoding forkhead transcription factor Foxo1 in rats with CCl_4_-induced ALI compared to the control animals. At the same time, the imbalance in the antioxidant system functioning caused by the GT activity suppression in CCl_4_ administration has been observed. It seems that the maladaptation expressed by the GT inhibition arises at the moment of the withdrawal of the animals from the experiment after CCl_4_ administration.

In our study, we observed hepatoprotective effects of DEQ treatment during the CCl_4_-induced ALI manifested in a decrease in the activities of ALT and AST in the serum as well as reduction of histological alterations in the liver, such as tissue necrosis, changes in liver architecture. Previous studies revealed that the mechanisms of hepatoprotective effects of DEQ were at least partly based on the reduction of CCl_4_-induced oxidative stress [29]. DEQ treatment reduced the intensity of CCl_4_-induced changes in the antioxidant system of rats. The studied substance decreased the gene expression and activity of AEs, such as CAT, SOD, GPX, and GR, and the gene expression of transcription factors, such as NF-κB2 and Nrf2 in the liver of rats with CCl_4_-induced ALI. Our data are in line with the results of previous studies showing the reduction in the intensity of free radical oxidation and restoration of activity of aconitate hydratase and NADPH-generating enzymes in the liver of rodents with CCl_4_-induced liver injury [51]. The DEQ-induced reduction in the intensity of CCl_4_-induced changes in oxidative stress might at least partly determine the hepatoprotective properties of this agent.

MPO is a well-known enzyme, primarily released by the activated neutrophils, which promotes the production of ROS and reactive nitrogen species in the cells and plays an important role in the pathogenesis of several liver disorders [52]. In our study, the CCl_4_-induced ALI in rats was accompanied by an increase in liver MPO activity reflecting enhanced neutrophil infiltration [53]. Interestingly, DEQ treatment significantly reduced an increase in this parameter in rats with modeled ALI. The mechanism of DEQ-induced reduction in MPO serum activity might be related to the direct inhibitory effects of this substance on the enzyme. It is known that some aromatic hydroxamates are able to competitively inhibit MPO by binding to the iron in the enzyme’s active center [54]. It might be also suggested that the formation of hydrogen bonds between oxygen molecule in the hydroxyl group of DEQ and residues of heme and arginine in the MPO active center contribute to the inhibitory effects of the study substance.

Inflammation is another crucial mechanism of ALI pathogenesis. Under ALI conditions, activated Kupffer cells, the resident macrophages of the liver, release pro-inflammatory cytokines, such as TNF-α and IL-1β. Previous data showed a positive correlation between TNF-α and IL-1β levels and markers of liver necrosis (ALT, and AST) in blood serum [55]. Therefore, the inhibition of release of pro-inflammatory cytokines is proposed as a possible therapeutic strategy for the treatment of inflammation and damage of the liver during ALI [56]. We showed that CCl_4_ administration is associated with an increase in the gene expression of pro-inflammatory cytokines IL-1β, IL-6 and TNF-α in the liver of rats. DEQ administration decreased the CCl_4_-induced increase in the expression of these cytokines, thus reducing the severity of developed hepatotoxicity. This might be considered as one of the mechanisms of hepatoprotective effects of this substance.

It was previously observed that CCl_4_ promotes the expression of inflammatory genes by the activation of the IKK (IκB kinase) complex via the toll-like receptor 4-mediated signaling pathway. This process is followed by the translocation of NF-κB to the nucleus, where it regulates the expression of inflammatory genes including the *Nfkb2* gene encoding NF-κB precursor p100 [57,58]. Therefore, suppression of the NF-κB signaling pathway is proposed as one of the primary mechanisms of pathogenesis of liver inflammation and associated diseases. We observed that the treatment with DEQ reduced the expression of *Nfkb2* gene in rats with ALI, which might mediate the observed reduction in the expression of pro-inflammatory cytokine genes.

ALI induced by peroral CCl_4_ administration was also previously shown to induce an increase in the *Ptgs2* transcription. Cyclooxygenase 2 is an enzyme degrading arachidonic acid to oxidized fatty acids, so-called prostaglandins. In addition to other functions, these fatty acids promote inflammation and portal hypertension [59]. Moreover, cyclooxygenase 2 is shown to regulate the activation of the NLRP3 inflammasome, one of the important mechanisms of CCl_4_-induced ALI. It is shown that NLRP3-inflammasome activation by CCl_4_ is associated with liver neutrophil infiltration and enhancement of ROS production, which aggravate ALI development [60]. Similar to the literature data, we observed that CCl_4_-induced ALI is accompanied by an increase in the NLRP3 protein level and activity of caspase-1. These processes might contribute to the maturation of proIL-1β to IL-1β and following increase in the concentration of IL-1β, which is, as discussed above, one of the main cytokines determining the innate and adaptive immune responses of organism to CCl_4_ [60].

Our study, for the first time, revealed that DEQ might suppress the activation of NLRP3 inflammasome during CCl_4_-induced ALI in rats. The administration of DEQ to rats with modeled ALI resulted in a decrease in the level of NLRP3 and IL-1β proteins, and activity of caspase-1 in the liver. Moreover, DEQ was shown to reduce MPO activity in the liver of rats with ALI comparing to the animals treated with CCl_4_ but not DEQ. These changes might be followed by a decrease in neutrophilic infiltration, and thus determine the inhibition of ROS generation during ALI. Interestingly, we showed the inhibitory influence of DEQ on the *Ptgs2* mRNA expression. This reduction might be related to the effects of DEQ on the lipid peroxidase activity, which was previously predicted to be suppressed by this agent with the probability of 0.697. Lipid peroxidase is one of the two active centers of the cyclooxygenase 2 catalytic domain, which contributes to the regulation of biosynthesis of a wide range of hydroperoxides and prostaglandins [61]. Another possible pathway of DEQ-induced inhibition of cyclooxygenase 2 activity might be associated with the reduction of expression of pro-inflammatory cytokines induced by this substance [62]. Altogether, these data might be considered as a possible mechanism of anti-inflammatory and antioxidant effects of DEQ observed in our study. We propose that the inhibition of NLRP3 inflammasome activation is one of the main mechanisms of hepatoprotective effects of DEQ during ALI.

Recent studies revealed a few direct and indirect NLRP3 inflammasome inhibitors, such as glyburide and its derivates 16673-34-0, FC11A-2, Parthenolide, VX-740, and oridonin. Some of these substances are used for a treatment of different inflammatory diseases, particularly type 2 diabetes mellitus, while the effects of others are still being studied. Some of these molecules possess low solubility and bioavailability, VX-740 is shown to cause hepatotoxicity, and oridonin might induce idiosyncratic toxicity and immune-mediated drug hypersensitivity. Glyburide can be used only for patients with diabetes due to pronounced hypoglycemic effect [63]. 

Apoptosis is a prominent feature of several liver diseases of various etiology including ALI. Mechanisms of hepatic apoptosis during liver injuries are determined by multiple signaling pathways and are dependent on the intensification of lipid peroxidation, endoplasmic reticulum stress, and involvement of innate and adaptive immune processes [64,65]. Our experiments revealed that the pathogenesis of CCl_4_-induced ALI is associated with significantly increased activity of caspase-3 and -8 in the liver of rats. Caspase-3 is a well-known mediator of apoptotic cell death, which determines the cleavage of several critical nuclear targets in the apoptotic cascade, such as caspase-activated deoxynuclease inhibitor and poly (ADP-ribose) polymerase (PARP) [66]. In turn, the activation of caspase-8 is required for efficient cleavage of caspase-3 and further initiation of apoptosis [67]. Previous data confirm our results showing the importance of hepatocyte apoptosis for the pathogenesis of CCl_4_-induced acute liver damage. Interestingly, the inhibition of caspase-3 followed by reduced PARP cleavage might be considered as a possible pathway for the protection of the liver from apoptosis [66]. Our study has revealed possible anti-apoptotic properties of DEQ under ALI conditions manifested in a significant decrease in the activities of caspases-3 and 8 in the liver of rats with CCl_4_-induced ALI. This mechanism might at least partly explain the hepatoprotective effects of the study substance during acute liver damage. It might be proposed that DEQ-induced reduction in the activities of caspases-3 and -8 is related to the suppression of the CCl_4_-induced oxidative stress as mentioned above [68]. Moreover, it is known that cytokines, particularly TNF-α and IL-1β, might activate the crucial members of programmed cell death, such as c-Jun N-terminal kinase (JNK) and P38 mitogen-activated protein kinase (MAPK) [69]. We showed that DEQ might reduce the expression of these pro-inflammatory cytokines, which is significantly enhanced during CCl_4_-induced ALI, and thus suppress the development of apoptosis.

Another possible mechanism of apoptosis during CCl_4_-induced liver injury is related to an increase in the expression of AIF, a mitochondrial inter-membrane space protein. After activation, AIF is getting translocated from the mitochondria to the nucleus during the mitochondrial outer membrane permeabilization, where it might induce double-stranded DNA breaks and a large-scale DNA condensation [70,71]. Previous data showed AIF to be involved in the pathogenesis of drug-induced liver injury [72]. In our study, an increase in *Aifm1* mRNA expression during CCl_4_-induced ALI was significantly reduced by DEQ. We propose that DEQ promotes the inhibition of both mitochondrial and ligand-mediated apoptosis pathways signaling, which might determine the anti-apoptotic and hepatoprotective properties of the studied substance.

**Figure 8:**
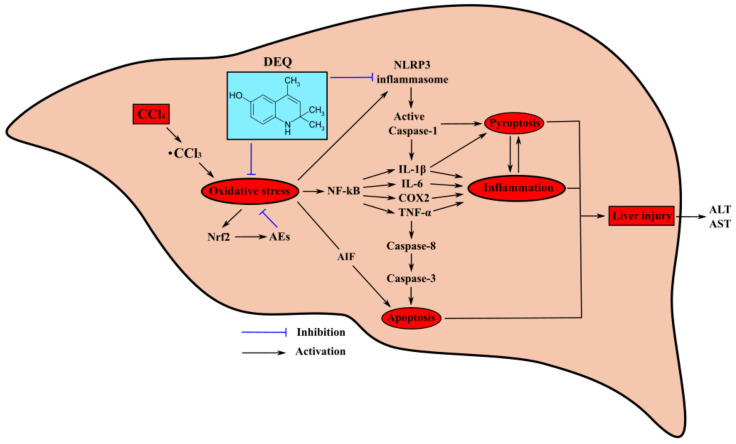
Schematic illustration of DEQ-mediated protective effects against CCl_4_-induced hepatotoxicity. Reactive oxygen species (ROS) formation during CCl_4_ administration induces hepatic pyroptosis and apoptosis via the activation of NLRP3 inflammasome, pro-inflammatory enzymes, and cytokines. Hepatoprotective effects of DEQ are based on the suppression of intensification of oxidative stress and activation of NLRP3 inflammasome through several cascades of molecular processes sufficient to improve acute injury outcomes of CCl_4_-induced hepatotoxicity.

The effects of DEQ on the two crucial pathogenetic pathways of ALI, such as intensification of oxidative stress and activation of NLRP3 inflammasome are shown in Figure 8. We suggest that hepatoprotective properties of this substance are based on the suppression of intensification of oxidative stress and activation of NLRP3 inflammasome through several cascades of molecular processes.

## 5. Conclusions

Altogether, our study revealed hepatoprotective properties of DEQ during CCl_4_-induced ALI. These effects of DEQ might be determined by its influence on the main pathogenetic pathways of this disorder, such as enhanced oxidative stress, intensified immune response manifested in the increased expression of pro-inflammatory mediators and cytokines, and intensified apoptosis in the liver. Thus, our results revealed a possible new therapeutic approach for ALI based on the hepatoprotective, anti-inflammatory, and antioxidant properties of DEQ observed on a rat animal model of liver injury.

## Figures and Tables

**Table 1:** The results of the prediction of biological activity spectra for substances (PASS) prediction study of the biological activities of deethylated ethoxyquin (DEQ).

Sl.no	Biological Activities	Deethylated Ethoxyquin
		Predicted LD50: 300 < LD50 ≤ 2000 mg/kg
		Toxicity Class: 4
		Pa
1	Reductant	0.888
2	Cytochrome P450 stimulant	0.870
3	CYP2C12 substrate	0.823
4	Progesterone antagonist	0.791
5	Chlordecone reductase inhibitor	0.790
6	Autoimmune disorders treatment	0.737
9	Membrane integrity agonist	0.736
10	CYP2J substrate	0.725
7	Kidney function stimulant	0.707
8	Lipid peroxidase inhibitor	0.697
13	CYP2J2 substrate	0.645
11	Radiosensitizer	0.642
12	Alkane 1-monooxygenase inhibitor	0.628
15	Lysase inhibitor	0.616
14	Glucocorticoid antagonist	0.593
23	Anti-inflammatory	0.583
16	Chemosensitizer	0.578
17	Menopausal disorders treatment	0.575
21	CYP2F1 substrate	0.572
22	MAP kinase stimulant	0.571
18	Free radical scavenger	0.567
19	Ophthalmic drug	0.565
20	Estrogen receptor beta antagonist	0.561

## Data Availability

Data is contained within the article

## Appendix A

The list of primers used in this work.

*Nfkb2* F: 5’- GAATTCAGCCCCTCCATTG-3′ and

*Nfkb2* R: 5′- CTGAAGCCTCGCTGTTTAGG-3′

*Il1b* F: 5′-TGTGATGAAAGACGGCACAC -3′ and

*Il1b* R: 5′-CTTCTTCTTTGGGTATTGTTTGG-3′

*Il6* F: 5′-CCTGGAGTTTGTGAAGAACAACT-3′ and

*Il6* R: 5′-GGAAGTTGGGGTAGGAAGGA-3′

*Tnf* F: 5′-TCTGTGCCTCAGCCTCTTCT-3′ and

*Tnf* R: 5′-GGCCATGGAACTGATGAGA-3′

*Ptgs2* F: 5′-TACACCAGGGCCCTTCCT-3′ and

*Ptgs2* R: 5′-TCCAGAACTTCTTTTGAATCAGG-3′

*Aifm1* F: 5′- AGTCCTTATTGTGGGCTTATCAAC-3′ and

*Aifm1* R: 5′- TTGGTCTTCTTTAATAGTCTTGTAGGC-3′

*Sod1* F: 5′-CCAGCGGATGAAGAGAGG-3′ and

*Sod1* R: 5′-GGACACATTGGCCACACC-3′

*Cat* F: 5′-CAGCGACCAGATGAAGCA-3′ and

*Cat* R: 5′-GGTCAGGACATCGGGTTTC-3′

*Nfe2l2* F: 5′-GCCTTGTACTTTGAAGACTGTATGC-3′ and

*Nfe2l2* R: 5′-GCAAGCGACTGAAATGTAGGT-3′

*Foxo1* F: 5′-AGATCTACGAGTGGATGGTGAAGAG-3′ and

*Foxo1* R: 5′-GGACAGATTGTGGCGAATTGAAT-3′

*Gsta2* F: 5′-CGGGAATTTGATGTTTGACC-3′ and

*Gsta2* R: 5′-AGAATGGCTCTGGTCTGTGC-3′

*Gpx1* F: 5′-TTTCCCGTGCAATCAGTTC-3′ and

*Gpx1* R: 5′-GGACATACTTGAGGGAATTCAGA-3′

*Gsr* F: 5′-TTCCTCATGAGAACCAGATCC-3′ and

*Gsr* R: 5′-CTGAAAGAACCCATCACTGGT-3′

*Casp3* F: 5′-GTGGAACTGACGATGATATGGC-3′ and

*Casp3* R: 5′-CGCAAAGTGACTGGATGAACC-3′

*Casp8* F: 5′-CTGGGAAGGATCGACGATTA-3′ and

*Casp8* R: 5′-CATGTCCTGCATTTTGATGG-3′

*Bcl2* F: 5′- GAGGATTGTGGCCTTCTTTG-3′ and

*Bcl2* R: 5′- AGGTACTCAGTCATCCACA-3′

*Bax* F: 5′- ATGGAGCTGCAGAGGATGA-3′ and

*Bax* R: 5′- CCAGTTTGCTAGCAAAGTAG-3′

*Gapdh* F: 5′-CCCTCAAGATTGTCAGCAATG-3′ and

*Gapdh* R: 5′-AGTTGTCATGGATGACCTTGG-3′
